# Couples Therapy Delivered Through Videoconferencing: Effects on Relationship Outcomes, Mental Health and the Therapeutic Alliance

**DOI:** 10.3389/fpsyg.2021.773030

**Published:** 2022-02-04

**Authors:** Andrea Kysely, Brian Bishop, Robert Thomas Kane, Maryanne McDevitt, Mia De Palma, Rosanna Rooney

**Affiliations:** School of Population Health, Faculty of Health Sciences, Curtin University, Perth, WA, Australia

**Keywords:** therapeutic alliance, couples therapy, videoconferencing, Couple CARE program, relationship satisfaction, mental health

## Abstract

Changing technology, and the pervasive demand created by a greater need in the population for access to mental health interventions, has led to the development of technologies that are shifting the traditional way in which therapy is provided. This study investigated the efficacy of a behavioral couples therapy program conducted *via* videoconferencing, as compared to face-to-face. There were 60 participants, in couples, ranging in age from 21 to 69 years old. Couples had been in a relationship for between 1 to 49 years. The 30 couples were randomly allocated to the face-to-face or videoconferencing group. They all took part in Couple CARE—a couples behavioral education program which promotes self-change in order to increase relationship satisfaction. The six session manualized intervention was offered in an identical manner to all clients, in each condition. Data analysis was based on several questionnaires completed by each couple at pre, post and 3-months follow-up. Results showed that therapeutic alliance ratings did not differ between groups, but increased significantly over time for both groups. Additionally, the results indicated improvements in relationship satisfaction, mental health, and all other outcome scores over time, which did not differ based on condition. This study uniquely contributes to the literature exploring the use of technology to provide therapy. Specifically, the study provides evidence for couples therapy *via* videoconferencing as a viable alternative to face-to-face interventions, especially for those couples who may not have access to the treatment they require. It is anticipated that the results of this study will contribute to the field of online therapy, and add to fostering confidence in agencies to allow expansion of services conducted *via* videoconferencing.

## Introduction

### The Use of Technology in Mental Health Services

Historically, psychological therapy has been conducted face to face, and rarely with the assistance of technology (Lungu and Sun, 2016). However, the rising need for access to mental health services, as well as the increased availability of the internet, have led to the development of new avenues through which psychological services can be delivered (Castelnuovo et al., 2003; Australian Government, 2012; Stubbings et al., 2015). The use of technology to provide mental health services is particularly valuable in Australia, where wide distances present a challenge for people living in rural and remote areas to access services (Richardson et al., 2009).

Tele-psychotherapy refers to therapy which is conducted at a distance, in contrast to the traditional face-to-face interaction in a therapist’s office (Oberkirch, 2002). Online therapy is a branch of tele-psychotherapy, which consists of mental health services that are provided through the use of the internet (Rees and Haythornthwaite, 2004). Tele-psychotherapy can be used for assessment, diagnosis, education and intervention (Rees and Haythornthwaite, 2004). Researchers have begun to investigate the merits of tele-psychotherapy interventions (Barak et al., 2008; Kraus, 2011). In its infancy, this research has focused on brief, simplistic, online methods such as emailing or text (Griffiths and Christensen, 2006). Although many studies have demonstrated the effectiveness of such interventions, the main argument against using these forms of therapy has remained, being that the absence of verbal cues in these simplistic forms hinders the effectiveness of the therapy (Griffiths and Christensen, 2006).

Of the numerous forms of tele-psychotherapy, videoconferencing is the closest to real world interaction, as it enables both verbal and visual cues to be transmitted between users (Castelnuovo et al., 2014; Deane et al., 2015). Unlike forms such as email, videoconferencing has the advantage of providing non-verbal feedback in real time. Non-verbal feedback from clients is an essential tool for therapists to track the effectiveness of their conversation (Caspar, 2005; Pepping et al., 2015). A client’s posture, tone, appearance, eye contact and verbal pace are often viewed as essential in allowing the therapist to regulate their intervention, and are particularly important in initial assessment. Non-verbal feedback from the therapist is also important for the client. Especially for clients of different cultures, non-verbal communication is significant in enabling trust to be established, and to demonstrate to the client that the therapist is sensitive and capable of understanding their unique worldviews (Sue and Sue, 2003).

The use of videoconferencing for therapeutic work has been a topic of research for over 50 years, with findings consistently reflecting high satisfaction rates, strong efficacy when compared to face-to-face therapy, and positive outcomes on clinical measures (Backhaus et al., 2012; Andersson et al., 2019; Keller et al., 2021).

Some researchers have aimed to engage the gold standard of research—the randomized controlled trial—by assigning participants to a face-to-face condition or a videoconferencing condition with a “treatment as usual” approach (Stubbings et al., 2013; Dear et al., 2015; Keller et al., 2021). The results of such studies have consistently identified few significant differences between conditions, indicating high satisfaction rates with the videoconferencing medium, comparable outcomes in terms of symptom reduction, adherence to treatment and engagement, as well as strong therapeutic alliances formed (Duncan et al., 2014). Alternatively, some studies have found that face-to-face therapy has been more effective than online therapy in forming the therapeutic alliance (Mallen et al., 2005). Research indicates that although videoconferencing contributes unique features as opposed to face-to-face therapy, such as a shared virtual environment, it is the therapeutic approach, rather than the technological medium, that has the largest impact on the therapeutic relationship developed (Cipolletta et al., 2018). This suggests that therapy has the same capacity to produce change when conducted online. Outcomes may also vary based on the form of therapy and the context in which it is used.

In Australia, where the population is geographically sparse, with over half a million people living in remote areas (National Rural Health Alliance, 2015), the need for such an initiative is growing (Oakes et al., 2007). With significant shortages of specialist psychologists in rural and regional areas, the alternative of videoconferencing to provide evidence based interventions has become a possible solution for many (Nelson et al., 2011; Richardson et al., 2015). Factors that may hinder the use of videoconferencing include the monitor initially being perceived as a barrier, and initial anxiety about the use of a new medium. Some clients might struggle due to having a lack of experience in using technology (Jang-Jaccard et al., 2014). However, these factors are amenable to change, and support can be provided to assist individuals who may not be as familiar with the medium (Morgan et al., 2008).

More recent years have seen a promising increase in studies examining the use of videoconferencing in the provision of psychological interventions. However, despite the increasing evidence of the efficacy of videoconferencing, there remain gaps in the literature base, including research on the use of specific interventions, their adaptation to online mediums with specific populations, the variety of psychological issues targeted, and direct comparisons using randomized control trials (Backhaus et al., 2012). A systematic review on videoconferencing psychotherapy found that individual and cognitive-behavioral interventions were the most commonly studied (Backhaus et al., 2012). While there has recently been an increase in studies examining online interventions aimed at couples, these interventions have typically been delivered using online content including videos and written material (Loew et al., 2012; Doss et al., 2020; Keller et al., 2021). A recent review has highlighted the lack of studies that have examined the use of videoconferencing as a means of delivering couples therapy (de Boer et al., 2021). In fact, of the 28 studies reviewed by de Boer et al. (2021), only 5 focused on videoconferencing with couples, while the remainder focused on family therapy with children and adolescents. Furthermore, of the randomized controlled trials that have been conducted, very few have compared online delivery with face-to-face delivery, with most studies choosing to compare the intervention group with a waitlisted control group (Loew et al., 2012; Doss et al., 2020; de Boer et al., 2021; Keller et al., 2021). This therefore presents a significant gap in the literature. Given differences between therapy with couples compared to individual therapy, the efficacy of videoconferencing as a medium for couples therapy is an area that warrants further investigation.

### The Need for Couples Therapy and Relationship Education

With a third of Australian marriages ending in divorce, and cohabitation relationships separating at a rate three to five times higher than marriages, relationship distress is seen as an increasingly central factor for couples engaging in relationship therapy. Relationship distress has a negative impact on an individual’s physical and mental health, and also affects others around them (Halford and Snyder, 2012; Rathgeber et al., 2019). Research has consistently shown that couples therapy effectively reduces relationship conflict and increases relationship satisfaction (Rathgeber et al., 2019). Furthermore, treatment with both individuals in the relationship has been found to be superior to individual treatment (Wilson et al., 2005).

As the scope of psychological intervention widens, the provision of services for couples to create strong, satisfying and resilient relationships has received more attention and resource allocation (Hawkins et al., 2008; Cicila et al., 2014). Couples can access help through both couples therapy and relationship education (Hawkins et al., 2012). Relationship education consists of two major components: the establishment of good communication and problem solving skills, and the provision of information (Hawkins et al., 2008). It is often manualized and preventative in nature (Halford and Snyder, 2012; Markman and Rhoades, 2012). Couples therapy is generally delivered to one couple at a time, when couples are already experiencing distress. Couple interventions can also combine features of both of these (Markman and Rhoades, 2012).

Despite the potential to engage in specialized interventions, and a steady increase in the availability of online therapy, couples therapy specifically appears to be lacking in research on tele-psychotherapy (Backhaus et al., 2012; de Boer et al., 2021). There is an increased demand for therapeutic intervention for couples, but a low supply of available services, creating a significant demand gap (Petch et al., 2014; Halford et al., 2015). In particular, research needs to explore the development of the therapeutic alliance between a therapist and couple online, as well as the effectiveness of specific manualized couple interventions (Markman and Rhoades, 2012). Research has already established that videoconferencing can be successful in a group setting, in terms of both outcome and process variables; however, few studies have specifically tested videoconferencing for couples therapy, which presents a unique dynamic between a therapist and a dyad (Greene et al., 2010).

### The Couple CARE Intervention

Couple CARE is a manualized program that focuses on behavior change in common areas of relationship functioning. It is an educational, skills-based tool that therapists can use in their work with couples, to alleviate distress and strengthen relationships (Halford et al., 2003). The program aims to evaluate each partner’s current behavior in specific domains, identify any goals for change, and then implement plans to reach those goals. Couple CARE is based on traditional behavioral couples therapy, and thus the content includes instruction, behavioral rehearsal, homework and feedback (Kelly and Iwamasa, 2005). The Couple CARE program is unique in that it allows couples to access and complete the majority of the program without necessarily entering the therapist’s office. Instead couples can engage in telephone sessions, in addition to work completed at home. The Couple CARE program represents a form of couples therapy that is particularly suitable for delivery over videoconferencing, as it is practical and skills-based in nature, focused on psychoeducation and behavior change.

Couple CARE has shown positive effects on relationship outcomes through several trials, which have been sustainable through follow-up periods (Halford and Bodenmann, 2013). Overall, the research shows the efficacy of the Couple CARE program in teaching couples important relationship skills such as effective listening and speaking skills, and caring behaviors to sustain mutually satisfying relationships in the long term (Halford et al., 2004, 2010; Petch et al., 2012). Additionally, the program has been shown to be equally effective when delivered face-to-face as well as in more flexible ways, such as with couples in their own dwelling, with regular telephone check-ins with the therapist (Halford et al., 2004, Halford and Bodenmann, 2013). Couple CARE also demonstrates high levels of participant satisfaction, as well as improvements in relationship contentment and durability (Halford et al., 2010).

The rationale of the current study was that there is a need for couples interventions that can be delivered *via* videoconferencing (Kysely, 2015). The aim of the study was to test the effectiveness of the Couple CARE program as delivered through videoconferencing (Kysely, 2015). Effectiveness was conceptualized in terms of relationship satisfaction and adjustment, desired and perceived change, mental health outcomes, marital happiness, therapeutic alliance ratings and participant satisfaction.

The effectiveness of the Couple CARE program was tested using a range of hypotheses. To directly test a null-hypothesis an unfeasible sample size would have been needed (Tabachnick and Fidell, 2007). Thus it was necessary to formulate hypotheses in terms of both treatments having positive effects, but face-to-face producing significantly better outcomes. Each hypothesis is therefore structured in two parts, addressing the effects of group (videoconferencing, face-to-face) and time (pre to post, pre to follow-up).

*Hypothesis 1a:* It is hypothesized that couple’s satisfaction and adjustment levels, as measured by improvement on the Dyadic Adjustment Scale (DAS), in the face-to-face condition, will be significantly different to the improvement scores on the DAS for couples in the videoconferencing condition. It is further hypothesized that this difference will reflect higher scores for participants in the face-to-face condition.
*Hypothesis 1b:* It is also hypothesized that there will be a statistically significant increase in dyadic adjustment as measured by the DAS from pre to post treatment, and this will be maintained at 3-month follow-up.

*Hypothesis 2a:* It is hypothesized that couples’ scores in the face-to-face condition on the Areas of Change Questionnaire (AC), in regards to desired change, will be significantly different to the couple’s scores on the AC in regards to desired change, for couples in the videoconferencing condition. It is hypothesized that this difference will reflect lower scores for couples in the face-to-face condition, reflecting less desired change from each respondent.
*Hypothesis 2b:* It is further hypothesized that there will be a statistically significant decrease in the amount of client’s desired change from their partners as measured by the AC from pre to post treatment, and that this will be maintained at 3 month follow-up.
*Hypothesis 2c:* It is similarly hypothesized that couples’ scores in the face-to-face condition on the AC in regards to the amount of change each participant perceives their partner requires, will be significantly different to those perceptions of couples in the videoconferencing condition. It is hypothesized that this difference will reflect lower scores, and thus less perceived change by those couples in the face-to-face condition.
*Hypothesis 2d:* It is also hypothesized that there will be a statistically significant decrease in the amount of change clients perceive their partners want them to engage in, as measured by the AC from pre to post treatment, and that this will be maintained at 3-month follow-up.

*Hypothesis 3a:* It is hypothesized that there will be a statistically significant difference in the scores on the DASS-42, for couples in the face-to-face condition, compared to those in the videoconferencing condition. It is hypothesized that this difference will reflect lower scores for depression, anxiety and stress symptoms of participants in the face-to-face condition, compared to those participants in the videoconferencing condition.
*Hypothesis 3b:* It is also hypothesized that there will be a significant decrease in depression, anxiety or stress scores as measured by the DASS-42 for participants following the intervention, and at 3-month follow up.

*Hypothesis 4a:* It is hypothesized that couples’ perceived alliance as measured by scores on the Working Alliance Inventory (WAI), will be significantly higher for couples in the face-to-face condition as compared to couples in the videoconferencing condition.
*Hypothesis 4b:* It is further hypothesized that there will be a statistically significant increase from session 3, to post intervention in client ratings of the working alliance as measured by the Working Alliance Inventory, and more specifically significant increases in the bond subscale.

*Hypothesis 5a:* It is hypothesized that relationship happiness as measured by the Marital Happiness Scale will be significantly greater in the face-to-face condition, in comparison to the videoconferencing condition.
*Hypothesis 5b:* It is also hypothesized that couples’ scores on the MHS will increase each week, as couples progress through the intervention.

*Hypothesis 6:* It is hypothesized that there will be a significant difference in the satisfaction scores of couples as measured by the Customer Satisfaction Questionnaire, with couples in the face-to-face condition reflecting higher satisfaction rates than those in the videoconferencing condition.

## Materials and Methods

### Research Design

The study used a randomized controlled trial methodology, with an active control group. Couples were randomly allocated to the face-to-face or videoconferencing condition, with the face-to-face condition being the active control group. The use of an active control allowed for a stronger test of the efficacy of videoconferencing, as the intervention had previously been established as efficacious and a comparison to a waitlist would allow for fewer conclusions to be made. In the videoconferencing condition both partners were in the same room, and the therapist in another room (replicating couples in rural areas accessing a therapist’s services in another location), and in the face-to-face condition, the couples were in the same room as the therapist (replicating couples in tradition face-to-face therapy). As the overall study used a mixed methods design, qualitative data was also collected, which is discussed in a separate paper (Kysely et al., 2020).

### Participants

**FIGURE 1:**
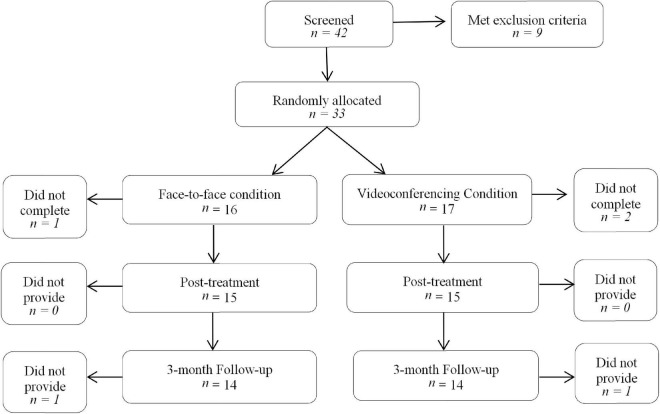
Participant flowchart. *n*, number of couples.

The sample included 33 couples who met the inclusion and exclusion criteria of the study. Inclusion criteria consisted of being over the age of 18, being in a defined *de facto* or marital relationship, and experiencing some mild relationship distress. Exclusion criteria included any risk of suicidal ideation, participation in a current couples therapy intervention, a DSM-IV diagnosis of psychosis or schizophrenia, severe alcohol/substance dependence, concurrent psychological treatment, or clinically significant relationship distress. During the study, three couples dropped out, leaving a total of 30 participating couples, with 15 in the intervention group and 15 in the control group, as displayed in Figure 1. Thus there were 60 individual participants involved. Participants’ ages ranged from 21 to 69 years, with a mean of 42.31 years. There were a variety of relationship lengths of couples, with couples being in a relationship from anywhere between 1 year to 49 years, with an overall mean length of relationship of 9.98 years. At 3-months follow-up, there were 28 couples still participating.

### Measures

**FIGURE 2:**
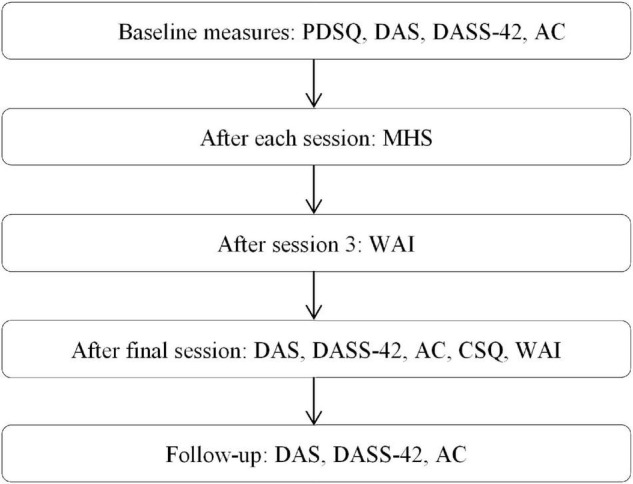
Flowchart of measures given to participants throughout the study. PDSQ, Psychiatric Diagnostic Screening Questionnaire, DAS, Dyadic Adjustment Scale, DASS-42, Depression, Anxiety and Stress Scale-42, Ac, Areas of Change Questionnaire, MHS, Marital Happiness Scale, WAI, Working Alliance Inventory.

Participants completed a number of questionnaires throughout the study. In addition, demographic information was collected at baseline. The measures completed at each time point are illustrated below (see Figure 2).

The Psychiatric Diagnostic Screening Questionnaire (PDSQ; Zimmerman and Mattia, 1999) is a self-report measure of psychopathology. It consists of 126 items that assess 13 DSM-IV diagnoses, as well as six additional items that screen for symptoms of psychosis. It takes 15–20 min to complete and is commonly given prior to the client’s first session. Participants are asked to rate their experience of an item within a specified time period, using a yes or no format. The PDSQ has moderate to high reliability; however, it is too sensitive in its diagnosis of alcohol dependence and abuse (Zimmerman and Mattia, 1999). If participants met this criteria, they completed a further follow-up phone interview using the appropriate section of the Mini International Neuropsychiatric Interview, to avoid misdiagnosis.

The Dyadic Adjustment Scale (DAS; Spanier, 1976) is a 32 item measure of relationship quality. Higher scores indicate higher dyadic adjustment. Questions reflect a general satisfaction or distress in a number of key relationship areas such as time spent together, communication, and future aims and goals. Furthermore four subscales—dyadic consensus, dyadic satisfaction, dyadic cohesion, and affection—can be used to gather more information about a certain relationship domain. Spanier’s (1976) initial study found strong internal consistency, with a test-retest coefficient of 0.96 for the total scale.

The Areas of Change Questionnaire (AC; Margolin et al., 1983) is used to assess each partner’s presenting complaints in important relationship domains such as intimacy, household duties, and time spent together or apart. It provides information on what type of changes the partner hopes to see in their spouse, and the extent to which each partner correctly defines the specific criteria the other wants changed. Partners are asked to rate on a scale of “much less” to “much more” how much they would like the area changed, or perceive their partner would like them to change. In the current study Areas of Change had an alpha of 0.87 and Areas of Perceived Change had an alpha of 0.89.

The Working Alliance Inventory (WAI; Horvath and Greenberg, 1989) assesses the quality of the therapeutic alliance between therapist and client. The WAI is a 36 item questionnaire that measures three domains of the working alliance: the emotional bond between the therapist and client, the importance placed on the goals of therapy, and the quality of the involvement both the therapist and client have in tasks in therapy. An overall score is also obtained of the amount of synchronicity across domains. Clients are asked to rate on a scale of “never,” to “always,” a variety of areas such as how much they perceive the therapist cares about them, how much they agree with the goals of the therapy, and how much they value the sessions.

The Depression, Anxiety and Stress Scales (DASS-42; Lovibond and Lovibond, 1995) are a collection of three self-report scales used to measure depression, anxiety and negative stress states during the past week. The scale requires individuals to rate symptoms on a four point Likert scale ranging from “did not apply to me at all” to “applied to me very much, or most of the time.” Higher scores reflect higher symptomology. Antony et al. (1998) found that the depression, anxiety and stress subscales had high internal consistencies and moderately high concurrent validity.

The Client Satisfaction Questionnaire (CSQ-8; Attkisson and Zwick, 1982) is a shorter alternative version of the original 31 item measure. The questionnaire is usually given to participants at the conclusion of an intervention, and asks about their opinions and any conclusions they may have made about their experience of the service. Responses are placed on a four point scale with total scores ranging between 8 and 32. Internal consistency ranges from 0.83 to 0.93; therefore the CSQ-8 is a shorter instrument that retains sound psychometric properties (Attkisson and Greenfield, 2004).

The Marital Happiness Scale (MHS; Azrin et al., 1973) estimates the amount of happiness or satisfaction each member of the couple feel in regards to their relationship. They are asked to mark on a scale of zero (completely unhappy) to 10 (completely happy) areas of a relationship such as household responsibilities, sex and personal independence. The scale also contained a “general happiness” question, which allowed for consistent overall comparison if individuals had not completed the entire questionnaire. The MHS allows for a valid weekly measure to be taken by couples, to mark potential progress throughout the intervention (Fowers and Olson, 1993). Couples were meant to complete this measure weekly as part of the study; however, many did not do so due to time restraints and forgetting to do so. Therefore, changes were tracked where possible, and corroborated through further qualitative check-ins with couples on a weekly basis.

### Equipment

The current study implemented the use of two Apple Mac computers, one which was placed in the therapist’s office, and one which was kept stationary for all couples to use. Both of these were in the Curtin Psychology Clinic, but in different rooms. The only program that was utilized for sessions was the iChat program which allowed for the therapist and clients to connect and both see and hear each other in real time, and for the therapist to record all sessions. The therapist would organize all connections prior to session, thus allowing the program to be available when couples entered the room. Whilst the therapist could see both the couple and themselves, the couples could only see the therapist, as not to distract or enable the participants to focus on their self-image.

### Procedure

Prior to the commencement of the study, ethics clearance was sought and provided by the Curtin Research Ethics Committee. Couples were recruited through the university and wider community, usually mediums such as newspaper, radio, mail, notice boards and online platforms. Once participants registered interest, they received a preliminary telephone call. If they remained interested and were suitable for the study, an information letter and consent forms were sent to their address. Once the consent forms were returned, an initial battery of tests and screening material was sent out. Once the complete clinical measures were received, scored, and found suitable for the study, participants were again contacted and given an appointment time as well as further information. Participants who met suicidal or psychotic criteria on the PDSQ, or who displayed significantly high distress rates on the DAS, were provided with referral numbers and the opportunity to meet with the project supervisors. It was explained to them that, as the program may not be appropriate to address their needs, it could not ethically be recommended to them.

Prior to the first session, couples were allocated to the videoconferencing or face-to-face condition using random allocation software. Couples in both groups were sent maps with directions to the psychology clinic. Couples in the face-to-face condition were shown to their rooms by the reception staff during office hours, and after-hours couples were greeted by the therapist in the reception room. Couples in the videoconferencing condition were informed over the phone that they would either be shown to the room by reception, and the therapist would appear on screen once they entered the room, or if after-hours appointments were scheduled, there would be signs posted on the building to show them the correct location of the clinic, upon entering which, they could go straight into the directed room. The therapist would then appear on screen and the session would begin.

The first half of the initial session was used to explain the study to the participants, and ensure they had read and understood the consent forms. After this, couples continued to work through all six sessions of the Couple CARE manual, with additional time provided to address any individual issues that may have arisen. During the intervention, participants filled out questionnaires at the allocated time points, as detailed previously (see Figure 2). Three months after the final session, all couples were contacted to participate in the completion of a final lot of questionnaires. The return of these completed each couple’s participation, and feedback about any changes evidenced in these was made available upon request.

### Intervention

Couple CARE is a manualized intervention comprised of six units covering topics of self-change, communication, intimacy and caring, managing differences, sexuality and adapting to change (Halford et al., 2004). These are all common areas of relationship functioning, and influence relationship satisfaction and distress. Couples are asked to watch a short segment on a DVD accompanying the program, and then complete a series of tasks individually and together throughout the week. Tasks are then evaluated each week with the help of the psychologist or counselor (Halford et al., 2004). This most commonly takes the form of self-change plans, where the participant is asked to come up with a task to enhance the area of relationship functioning in review that week. The role of the therapist is to not only review how successful they were in completing their self-change plans, but also to demonstrate and discuss the concepts pertinent to that week’s relationship area. Furthermore, their role includes helping the couples to then implement their plan, and troubleshoot any difficulties that may arise in completing their tasks. The time frame of completing the program is 6 weeks, with sessions taking place once a week, and couples watching the DVD and completing tasks in their own time, between sessions.

### Analysis

Generalized linear mixed models (GLMM) was the most appropriate analysis for the dyadic data in this study. A GLMM is a special class of regression model and an extension to the generalized linear model, in which the “mixed” linear predictors contains random effects in addition to fixed effects (Breslow and Clayton, 1993). The generalized element of the regression model allows it to accommodate outcome variables with non-normal distributions, such as ordinal data with restricted ranges, binary variables, proportions, and count data. The current study employs a research design that has two nominal random effects, being the dyad and the participants within the dyad, one categorical fixed effect, being the condition (face-to-face, videoconferencing), and finally one ordinal fixed effect of time (pre, post, follow-up). Because of this, GLMM, implemented through SPSS, was ideally suited for analyzing this set of data, and was able to analyze the effects of both group and time (Breslow and Clayton, 1993). GLMM also accounts for participant attrition and intra-couple clustering in the data (Hartzel et al., 2001).

Group × Time interaction effects were tested for each outcome. If the Group × Time interaction was non-significant, this meant that both main effects could be interpreted independently. In addition, the Group × Time interaction embodies the intervention effect (i.e., the difference between the face-to-face and videoconferencing conditions. Follow up analyses for the main effects of time were conducted using *post hoc* LSD (least significant difference) contrasts as implemented through the GLMM.

### Statistical Power

Twenty-eight couples (the number of couples at follow-up), 14 in each condition, were sufficient for detecting moderate to large Group × Time interactions (*f* > 0.25) (Hemming et al., 2011). There was therefore insufficient power to detect smaller interactions, i.e., smaller differences between the face-to-face and videoconferencing conditions. This was not considered problematic since small effects are rarely of clinical importance.

## Results

### Relationship Adjustment and Satisfaction (DAS)

The Group × Time interaction was non-significant (*F*_2,170_ = 0.20, *p* = 0.821), as was the main effect of Group (*F*_1,170_ = 0.01, *p* = 0.938), indicating that couples’ relationship adjustment and satisfaction levels did not differ significantly between conditions at any time point. The analysis did not identify any significant differences between groups in the DAS subscales of cohesion (*F*_1,170_ = 0.017, *p* = 0.895), consensus, (*F*_1,170_ = 0.194, *p* = 0.660), satisfaction (*F*_1,170_ = 0.001, *p* = 0.973) or affection (*F*_1,170_ = 2.499, *p* = 0.116).

The main effect for Time was significant (*F*_2,170_ = 5.47, *p* = 0.005). Follow-up analyses indicated that, for both conditions, there was a significant pre-post increase on the DAS (*p* = 0.002), with a moderate effect size (η^2^ = 0.06). There was also a small, significant pre-FU increase across both conditions (*p* = 0.017, η^2^ = 0.03). Scores on the consensus, (*F*_2,170_ = 5.033, *p* = 0.008), satisfaction (*F*_2,170_ = 4.354, *p* = 0.014), and affection subscales (*F*_2,170_ = 3.060, *p* = 0.049) showed a significant difference between collection points, with scores increasing positively over time. The cohesion subscale was the only subscale not to show a significant increase in scores (*F*_2,170_ = 2.512, *p* = 0.084). Table 1 shows a detailed outline of the means and standard deviations of the DAS for each condition and time point, on all subscales.

**TABLE 1:** Means (adjusted means) and standard deviations for the dyadic adjustment scale and subscales, and the areas of change questionnaire subscales in the videoconferencing and control conditions (*N* = 60).

Outcome	Videoconferencing group		Face-to-face group	
	(*N* = 30)		(*N* = 30)	
	Mean	SD	Mean	SD
Pretest DAS Posttest DAS 3-month DAS	100.83 106.47 106.71 (107.13)*	11.77 12.87 14.46	100.83 107.33 105.71 (105.51)	11.46 13.90 15.39
Pretest DAS_Con Posttest DAS_Con 3-month DAS_Con	44.73 46.80 47.96 (48.29)	7.33 6.43 7.54	45.00 48.27 47.61 (47.30)	6.88 6.92 7.66
Pretest DAS_Coh Posttest DAS_Coh 3-month DAS_Coh	14.60 15.30 14.43 (14.46)	3.14 2.77 3.58	14.73 15.60 14.86 (14.90)	3.08 2.77 2.61
Pretest DAS_Sat Posttest DAS_Sat 3-month DAS_Sat	34.10 36.40 36.82 (36.85)	4.81 5.59 5.84	34.90 36.33 36.25 (36.25)	4.28 5.74 5.70
Pretest DAS_Aff Posttest DAS_Aff 3-month DAS_Aff	7.47 8.03 7.50 (7.54)	1.98 2.11 3.10	6.10 7.13 7.00 (7.00)	1.97 2.27 2.58
Pretest AC_Desired Posttest AC_Desired 3-month AC_Desired	19.00 14.77 15.50 (15.57)	11.68 10.41 10.48	19.40 17.47 15.37 (15.51)	12.09 11.93 8.97
Pretest AC_Perceived Posttest AC_Perceived 3-month AC_Perceived	21.17 16.83 16.03 (15.79)	10.33 9.26 9.11	25.40 20.03 18.74 (18.39)	14.64 14.47 11.82

*DAS, Dyadic Adjustment Scale; DAS_Con, Dyadic Adjustment Scale, Consensus Subscale; DAS_Coh, Dyadic Adjustment Scale, Cohesion Subscale; DAS_Sat, Dyadic Adjustment Scale, Satisfaction Subscale; DAS_Aff, Dyadic Adjustment Scale, Affection Subscale; AC_Desired, Areas of Change Questionnaire, Desired Change Subscale; AC_Perceived, Areas of Change Questionnaire, Perceived Change Subscale.*

*Missing values were recorded at follow-up, GLMM uses adjusted means when there are missing values and these are displayed in parentheses.

### Desired and Perceived Change (AC)

The Group × Time interaction was non-significant for the AC_Desired scale (*F*_2,169_ = 0.58, *p* = 0.564). The main effect of Group was also non-significant (*F*_1,169_ = 0.19, *p* = 0.665), indicating that there were no significant differences in desired change between conditions. The main effect of Time was significant for the AC_Desired scale (*F*_2,169_ = 4.40, *p* = 0.014). Follow-up analyses indicated that, for both conditions, there was a significant pre-post decrease in desired change (*p* = 0.041), with a small effect size (η^2^ = 0.02). Both conditions showed a small, significant pre-FU decrease (*p* = 0.004; η^2^ = 0.05) indicating that the effect was maintained at follow-up.

The Group × Time interaction was non-significant for the AC_Perceived scale (*F*_2,169_ = 0.08, *p* = 0.924), as was the main effect of Group (*F*_2,169_ = 1.44, *p* = 0.231), indicating that the conditions did not differ in perceived change at any time point. The main effect for Time was significant (*F*_2,169_ = 0.567, *p* = 0.004), and there was a moderate, significant pre-post decrease in perceived change (*p* = 0.001; η^2^ = 0.06) across both conditions. Both conditions also had a small, significant pre-FU decrease (*p* = 0.004; η^2^ = 0.05); meaning that the effect of the intervention was maintained at follow-up. Table 1 shows a more detailed outline of means and standard deviations for the AC in each condition and time point.

### Depression, Anxiety and Stress (DASS-42)

The Group × Time interaction was non-significant for the Depression (*F*_2,170_ = 0.44, *p* = 0.648), Anxiety (*F*_2,170_ = 0.79, *p* = 0.458), and Stress subscales (*F*_2,170_ = 0.17, *p* = 0.846). The main effect of Group was also non-significant for the Depression (*F*_1,170_ = 0.84, *p* = 0.361), Anxiety (*F*_1,170_ = 0.00, *p* = 0.973), and Stress subscales (*F*_1,170_ = 0.14, *p* = 0.713), indicating that there were no significant differences between groups in depression, anxiety or stress at post-test or at 3-months follow-up.

The main effect for Time was significant for the Depression (*F*_2,170_ = 12.40, *p* = 0.000, Anxiety (*F*_2,170_ = 5.05, *p* = 0.007), and Stress subscales (*F*_2,170_ = 11.18, *p* = 0.000). Follow-up analyses indicated that, for both groups, there was a moderate, significant pre-post decrease in depression (*p* = 0.000; η^2^ = 0.09). Neither group showed a significant pre-FU decrease (*p* = 0.076) indicating no maintenance of effects on depression at follow-up. Both groups had a small, significant decrease in anxiety at post-test (*p* = 0.003; η^2^ = 0.05) as well as at follow-up (*p* = 0.012; η^2^ = 0.04), suggesting a maintenance of the effects on anxiety. Finally, there was a moderate, significant pre-post decrease in stress scores for both groups (*p* = 0.000; η^2^ = 0.12). Both groups showed a significant pre-FU decrease (*p* = 0.000) suggesting a maintenance of the effects on stress at follow-up, with a moderate effect size (η^2^ = 0.08).

### Happiness (MHS)

The Group × Time interaction was non-significant for the MHS administered weekly to each couple (*F*_4,168_ = 0.21, *p* = 0.053). The main effect of Group was non-significant (*F*_1,168_ = 0.32, *p* = 0.572), indicating that the groups did not differ significantly on this outcome at any assessment points. The main effect for Time was significant (*F*_4,168_ = 9.01, *p* = 0.000). As evidenced in Table 2, follow-up analyses indicated that, for both groups, there was a small, significant increase on the MHS between session 2 and session 4 (*p* = 0.006; η^2^ = 0.04). Significant increases in scores were also found between session 3 and session 6 (*p* = 0.001), with a moderate effect size (η^2^ = 0.06), as well as a significant increase between session 4 and session 6 (*p* = 0.024), with a small effect size (η^2^ = 0.03). There were no significant increases between sessions 2 and 3 (*p* = 0.126), sessions 2 and 5 (*p* = 0.050), sessions 3 and 4 (*p* = 0.216), sessions 3 and 5 (*p* = 0.170), sessions 4 and 5 (*p* = 0.530), or sessions 5 and 6 (*p* = 0.155). Finally there was a moderate, significant increase between session 2 and session 6 (*p* = 0.006; η^2^ = 0.10), suggesting a maintenance of the effect for both conditions.

**TABLE 2:** *Post hoc* LSD contrasts of the marital happiness scale.

Contrast	Contrast estimate	Std. error	*t*	df	Adj. Sig.	95% Confidence	
						interval	
						Lower	Upper
S2-S3	−0.252	0.164	−1.538	168	0.126	−0.576	0.071
S2-S4	−0.458	0.165	−2.767	168	0.006	−0.784	–0.131
S2-S5	−0.582	0.295	−1.972	168	0.050	−1.165	0.001
S2-S6	−0.837	0.189	−4.432	168	0.000	−1.210	–0.464
S3-S4	−0.206	0.166	−1.241	168	0.216	−0.533	0.121
S3-S5	−0.330	0.240	−1.377	168	0.170	−0.803	0.143
S3-S6	−0.585	0.174	−3.362	168	0.001	−0.928	–0.241
S4-S5	−0.124	0.197	−0.630	168	0.530	−0.514	0.265
S4-S6	−0.379	0.166	−2.280	168	0.024	−0.708	–0.051
S5-S6	−0.255	0.179	−1.429	168	0.155	−0.607	0.097

### Working Alliance (WAI)

The Group × Time interaction was non-significant for the WAI (*F*_1,116_ = 0.21, *p* = 0.650). The main effect of Group was also non-significant (*F*_1,116_ = 0.55, *p* = 0.458), indicating that the groups did not differ significantly in perceived alliance ratings at the two assessment points. The main effect of Time was significant (*F*_1,116_ = 20.82, *p* = 0.000). There was a large, significant increase in WAI scores from Session 3 to Session 6 across both groups (*p* = 0.000; η^2^ = 0.15). It should be noted that no follow-up measures were collected for perceived alliance. Table 3 displays means and standard deviations for the WAI at both time points.

**TABLE 3:** Means and standard deviations for the working alliance inventory in the videoconferencing and control conditions (*N* = 60).

Outcome	Videoconferencing group		Face-to-face group	
	(*N* = 30)		(*N* = 30)	
	Mean	SD	Mean	SD
Session 3 WAI Session 6 WAI	194.63 202.77	7.81 8.11	202.17 212.10	8.11 8.49

*WAI, Working Alliance Inventory.*

Specifically for the WAI bond subscale, there was a non-significant Group × Time interaction (*F*_1,116_ = 0.00, *p* = 0.983) and a non-significant main effect of Group (*F*_1,116_ = 0.98, *p* = 0.324), indicating no difference between conditions. The main effect for time, however, was significant (*F*_1,116_ = 11.37, *p* = 0.001). Both groups displayed a moderate, significant increase in WAI bond scores over time (*p* = 0.001; η^2^ = 0.09).

### Participant Satisfaction

Data was only collected for the Client Satisfaction Questionnaire at one time point. The main effect of Group was non-significant (*F*_1,58_ = 0.045, *p* = 0.833), indicating that the conditions did not differ in participant satisfaction. This is confirmed by the similarity in scores between the face-to-face condition (*M* = 28.47, *SD* = 3.42), and the videoconferencing condition (*M* = 28.70, *SD* = 3.41).

## Discussion

The current study was able to demonstrate that videoconferencing is efficacious in connecting a couple and therapist, for the provision of couples intervention (Kysely, 2015). The results indicate success in the two largest areas of investigation in relation to online therapies, namely, therapy outcomes and satisfaction (Morgan et al., 2008). Furthermore, Couple CARE delivered through videoconferencing demonstrated comparable outcomes to Couple CARE delivered face-to-face.

Firstly, the Couple CARE intervention as delivered through videoconferencing was able to effect a number of relationship therapy outcomes, supporting a number of hypotheses. A post intervention increase in relationship adjustment and satisfaction was found across both conditions (H1b), which was maintained at follow-up. Results also indicated that couples in both conditions had less desired and perceived change (H2b; H2d) after the intervention and at follow-up. Effects were also seen on mental health outcomes (H3b). Depression decreased across both groups post intervention, but this was not maintained at follow up. A decrease was found in anxiety and stress for both groups; this result was found post intervention and also was maintained at 3-months follow-up. Thus, the hypothesis was mostly supported, except that the decreased in depression was not maintained. There was a positive, significant increase over time in relationship happiness (H5b), supporting the hypothesis. These findings align with those of previous studies, which have found online interventions to be successful in producing therapy outcomes (Barak et al., 2008; Greene et al., 2010; de Boer et al., 2021).

A number of hypotheses relating to the therapeutic outcomes (H1a; H2a; H2c; H3a; H5a) predicted that couples in the face-to-face condition would have better outcomes than couples in the videoconferencing condition. However, there were no significant differences between conditions in relationship satisfaction (H1a), desired and perceived change (H2a; H2c), mental health (H3a) or happiness (H5a). As none of these hypotheses pertaining to group differences were supported, it can be concluded that videoconferencing was as effective as face-to-face delivery. Given that there were no significant differences between groups and both groups showed positive changes in terms of relationship satisfaction and decreases in symptoms of depression, anxiety and stress, the results of this study suggest that videoconferencing is equally as effective as face-to-face therapy.

Secondly, efficacy was displayed in therapeutic process outcomes. It was predicted that perceived alliance (H4a) and participant satisfaction (H6) would be higher for couples in the face-to-face condition. However, results indicated that neither of these hypotheses were supported. This is a promising finding, as it means that videoconferencing was as effective as face-to-face in achieving these process outcomes. Furthermore, the perceived alliance ratings increased over time for both conditions (H4b), supporting the hypothesis and indicating that videoconferencing was as effective as face-to-face at building the therapeutic alliance.

Consistent with previous research (Stubbings et al., 2013), the current study was able to demonstrate that despite never seeing the therapist face-to-face, the videoconferencing couples identified the same levels of perceived alliance. Simpson et al. (2005) proposed that if a client feels strongly aligned to the therapist, the mode in which they connect becomes redundant, and significantly less problematic. In the current study, the initial alliance scores for participants in the videoconferencing condition were not significantly different to those in the face-to-face condition. Furthermore, as the study progressed, couples in the videoconferencing condition did not report significantly lower scores than those in the face-to-face condition, reflecting a strong alliance, despite physically being in an alternative location to that of the therapist. The current study evidences support for previous findings that reflect that an alliance can be established when using technology to facilitate therapy (Day and Schneider, 2002; Mallen et al., 2005; Morgan et al., 2008; Simpson and Reid, 2014; de Boer et al., 2021), and this is consistently true even in couples therapy. The data also reflected an overall satisfaction by couples with the program.

### Implications of the Study

This study has contributed to a very small body of research examining videoconferencing as a means for therapy with couples (de Boer et al., 2021). Up until recently, studies examining online therapy for couples have been lacking and the body of research specifically examining couple therapy *via* videoconferencing remains minimal (Backhaus et al., 2012; de Boer et al., 2021; Keller et al., 2021). Given the statistically significant, and overwhelmingly positive results gleamed from the data collected, this study could be a noteworthy piece of evidence for the expansion of services provided technologically. Videoconferencing provides individuals in rural, military, and Fly-in Fly-out (FIFO) contexts with the opportunity to connect with a therapist who may be more flexible, or appropriately skilled, to address the needs of that client.

The current study was able to evidence the ability of videoconferencing in making this connection effective and viable for these populations. Furthermore, the study was able to demonstrate that despite adding another client to the therapeutic intervention, in the form of “the partner,” strong rapport could be developed and substantial benefits gained from the intervention. This therefore leaves two main implications evident in the results of the current study. Firstly, that videoconferencing is a viable means of engaging clients that may otherwise not seek therapeutic intervention, and secondly, that a couples’ intervention specifically can be conveyed successfully through such a medium.

Given that historically some therapists have held reservations about the ability of videoconferencing to foster a successful and therapeutically relevant relationship (Rees and Stone, 2005), the results of the current study contribute to a growing body of research suggesting that the alliance established through videoconferencing compares to that of face-to-face therapy (Rees and Maclaine, 2015; Richardson et al., 2015; de Boer et al., 2021). The current study was able to demonstrate the ability to foster such an alliance with a couple, despite no physical contact. The results of this study could therefore be used to enhance expansion of services to couples through technology, and provide therapists with a sense of confidence of its efficacy. The results of the current study can be used to inform policy directions and therapeutic intervention planning, and in this way have real world implications for the provision of clinical interventions for couples.

### Strengths and Limitations

The current study involves a number of strengths, such as clear identification of the intervention’s aims and the use of validated measures. The measures utilized in the current study have shown high reliability and validity in previous studies (Weiss et al., 1973; Horvath and Greenberg, 1989; Fowers and Olson, 1993; Antony et al., 1998; Attkisson and Greenfield, 2004; Garbarini et al., 2014), and high internal consistency in the current study. Another strength was having a previously established intervention (Halford et al., 2004; Petch et al., 2012) that was standardized across conditions, allowing for greater comparability. A single therapist was also used to conduct all interventions; this minimized any therapist confounding variables.

Furthermore, another strength of the current study design was its use of sealed envelopes provided to couples for the return of their WAI questionnaires. This was to ensure participants did not feel a sense of pressure, or fear of negative reprisal from the therapist, based on their feedback. Participants were reassured that results would not be viewed until their completion of the program. In terms of follow-up, while some studies have had longer follow-up periods (Halford et al., 2007; Hahlweg and Richter, 2010), given the resources and timespan of the current study, the 3-month follow-up period was adequate in demonstrating significant pre to follow-up results.

Couples experiencing moderate to higher degrees of distress are often excluded from couples intervention research, limiting the generalizability of results (Halford et al., 2015; Pepping et al., 2015). Although clients experiencing higher degrees of distress were excluded from the current study due to ethical considerations, those who displayed mild-to-moderate distress were allowed to participate in this study, providing a wider range. This gives the study more depth and increased ability to generalize to real world populations.

One potential limitation was the sample size, which was not large enough to have sufficient power to detect smaller effects. However, it was sufficient to produce statistically significant results and moderate as well as small effects (Stubbings et al., 2013; Théberge-Lapointe et al., 2015). This was not considered problematic, since small differences between the two treatments on the present outcomes were considered to be of little clinical importance. Furthermore, the sample size used here compares well with other similar studies, and is actually larger than many (Jedlicka, 2001; Tambling and Johnson, 2010; Stubbings et al., 2013).

Another limitation was that, due to the experimental design, we were unable to test whether or not similar results would be produced in a less controlled but more natural context, such as couples accessing the therapy from their homes. The setting was chosen to minimize variables that may influence Couple CARE outcomes. By varying only whether the session was on video or face to face meant we were more confidently able to say the difference lay in the medium (i.e., face to face or *via* video) by which Couple CARE was delivered. Future research should address the additional variables that may influence Couple CARE outcomes such as whether video-conferencing takes place at home or not.

One criticism of telepsychology is the risk of engaging severely distressed clients, as the therapist is unable to respond to risks in person (Kramer et al., 2015). To minimize risk in this study, couples were screened and risk assessments were completed prior to their engagement in the intervention. It is of note that videoconferencing is actually able to prevent some risks, due to its technological nature. For example, as everything is recorded, this becomes an incentive for partners to maintain “good behavior,” otherwise evidence could be presented for prosecution of any violent or malicious acts. To further ensure safety, the possibility could be explored of having clearly defined protocols when working with clients in different locations, such as local support locations or emergency response personnel. A contract may need to be signed, designating a support person or emergency contact that could be contacted if needed to ensure the safety of the client.

### Future Directions

Having each member of the dyad in the same room may have helped build on the feeling of presence, and made it easier for couples to immerse themselves in the therapeutic process. Future studies could explore the effects on the dynamic where both partners are in separate locations, as well as the therapist. This would result in a scenario where three individuals are connected from separate locations. In this way couples could engage in therapy even if they are not in the same place (McCoy et al., 2013). Including couples that may be experiencing more significant levels of distress, or those facing issues such as drug or alcohol abuse, could further evidence the appropriateness of videoconferencing for couples intervention and, perhaps even more intensive therapy. Having a larger sample size would be useful to further explore comparisons between face-to-face and videoconferencing conditions, as this may show smaller discrepancies.

Furthermore, future research could also focus on varying interventions, ones that are perhaps more intensive, and less structured. The current study used a behavioral couples intervention. Again being one of the first studies of its kind, it was important to use a structured intervention that could truly be compared by exposing couples in both groups to the same intervention. However, it would be interesting to explore whether a more emotionally vested, intensive, less structured intervention such as Emotion Focused Therapy would be affected by the technological element of videoconferencing. In this way the question of whether the same depth of alliance could be established through videoconferencing as in the same physical room, could be further explored.

In terms of the structure of the study and data collection, limited studies have been produced that have longer follow-up periods available for comparison (Simpson and Reid, 2014). Therefore whilst studies such as the current one can show successful outcomes from a specific therapeutic intervention, more research is needed on whether there results can be sustained for longer periods of time. These results could then be further compared between conditions, and therapy that is presented through a technological medium.

In conclusion, this study has significantly contributed to the growing body of research reflecting the use of technology to facilitate therapeutic intervention. This study has assisted in demonstrating the validity and acceptability of the use of technology for couples therapy, which will ultimately expand services to various “in need” populations, and therefore assist in breaching the existing demand gap. In recent years the use of online therapies and technology itself has increased exponentially, with research finding that online therapeutic interventions can be as effective as traditional face-to-face therapy. Given the strong evidence base which the current study has contributed to, and the niche, yet significant market for couples intervention online, it is hoped that this growth continues. It is important to harness these gains, and use them to enhance peoples’ wellbeing everywhere.

## Data Availability Statement

The raw data supporting the conclusions of this article will be made available by the authors, without undue reservation.

## Ethics Statement

The studies involving human participants were reviewed and approved by Curtin University Human Research Ethics Committee. The patients/participants provided their written informed consent to participate in this study. Written informed consent was obtained from the individual(s) for the publication of any potentially identifiable images or data included in this article.

## Author Contributions

AK carried out and conceptualized the project, including running the intervention and analyzing the data, and wrote the research. BB contributed to the conceptualization of the project, supervised by AK during the project and contributed to the write up of the research. RK was involved in the conceptualization of the project, supervision of the analysis, and writing up of the research. MM contributed to the write up of the research manuscript. MD contributed to the write up of the research manuscript. RR supervised AK during the project, clinically supervised Couple CARE, and contributed to the write up of the research. All authors contributed to the article and approved the submitted version.

## Conflict of Interest

The authors declare that the research was conducted in the absence of any commercial or financial relationships that could be construed as a potential conflict of interest.

## Publisher’s Note

All claims expressed in this article are solely those of the authors and do not necessarily represent those of their affiliated organizations, or those of the publisher, the editors and the reviewers. Any product that may be evaluated in this article, or claim that may be made by its manufacturer, is not guaranteed or endorsed by the publisher.
